# Massive effects on chromatin after ploidy rearrangement in doubled haploids

**DOI:** 10.1093/jxb/erac478

**Published:** 2023-02-04

**Authors:** Eduardo Mateo-Bonmatí

**Affiliations:** Cell and Developmental Biology, John Innes Centre, Norwich Research Park, Colney Ln, NR4 7UH, UK

**Keywords:** Arabidopsis, chromatin, DNA methylation, doubled haploid, epigenetics, whole-genome duplication, whole-genome elimination

## Abstract

This article comments on:

**Piskorz EW, Xu L, Ma Y, Jiang H**. 2023. Doubled-haploid induction generates extensive differential DNA methylation in Arabidopsis. Journal of Experimental Botany **74**, 835–847

**Breeding programmes for major crops, such as maize, wheat, or rice, among others, use doubled-haploid (DH) induction as a strategy to speed up the process to obtain pure homozygous lines. DHs are obtained in two phases: the first one requires the creation of a haploid plant whose chromosomes will be doubled in a subsequent step. However, this constant playing with ploidy levels usually entails substantial changes in the chromatin environment. Now, Piskorz *et al*. (2023) have demonstrated that indeed both steps produce thousands of changes at the DNA methylation level, most of which occur randomly.**

Most eukaryotic species have a stable chromosome number that is faithfully maintained over generations. Complex machinery operates to keep constant ploidy levels to avoid potential harmful effects. Sexually reproducing organisms alternate two ploidy phases; in diploids, for instance, a diploid (two chromosome sets) phase in which both chromosomes are equally distributed and copied after mitosis, and a haploid phase (one set of chromosomes), when gametes are developed through meiosis. After fertilization, two single sets of chromosomes merge and give rise to a new diploid embryo.

The need to accurately control ploidy levels derives from the necessary control of gene dosage. Incorrect chromosome segregation can lead to excess or lack of a product of many genes, generally conflicting with cell viability. Several well-characterized human diseases are known to arise from monosomies (one chromosome) or trisomies (three chromosomes). The best understood example of naturally occurring gene dose control is the human X-chromosome inactivation in females (Plath *et al.*, 2002).

Eukaryotic chromosomes are formed by a macromolecular complex called chromatin composed of DNA wrapping octamers of proteins named histones. The ability of the transcriptional machinery to access a given chromatin section relies on the different chemical modifications, which include, among others, methylation on both histones and DNA (Rando and Chang, 2009) (Fig. 1A). In plants, DNA cytosines can be methylated in three different contexts: the symmetrical CG and CHG and the asymmetrical CHH (being H any nucleotide but G). In Arabidopsis, the DNA methyltransferase MET1 is mainly responsible for DNA methylation at the CG context, and non-CG methylation is mediated by CHROMOMETHYLASE (CMT) and DOMAINS REARRANGED METHYLTRANSFERASE (DRM) proteins (Zhang *et al.*, 2018).

**Fig. 1.**
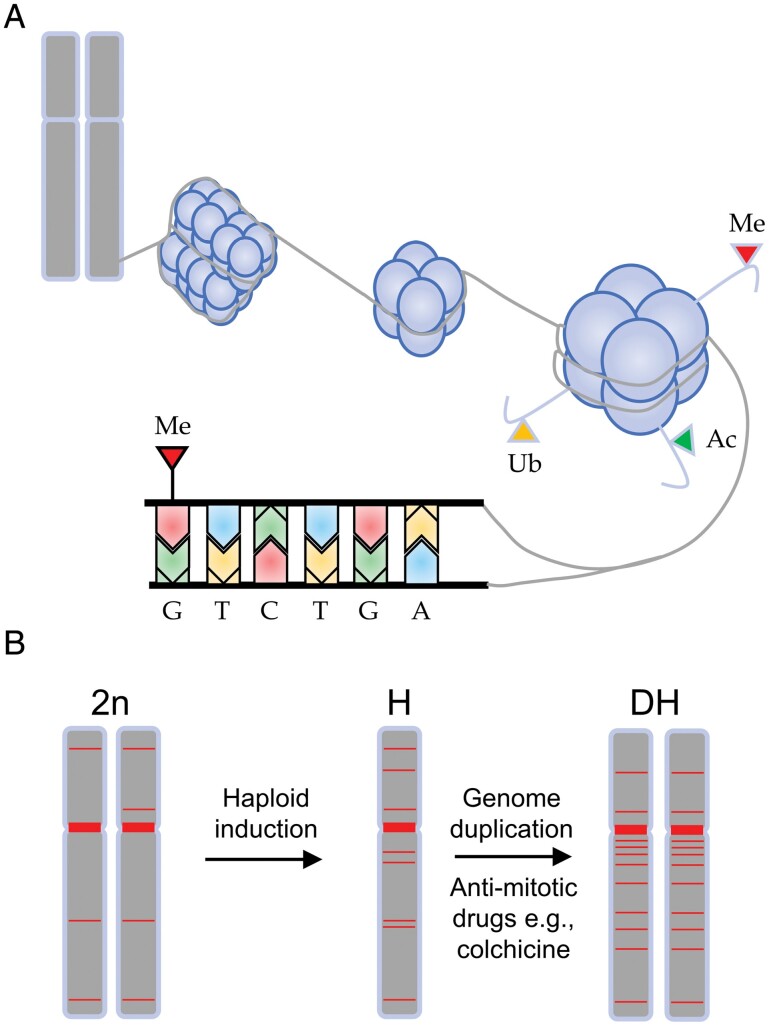
doubled-haploid (DH) induction generates massive stochastic DNA methylation. (A) Chromosomes are formed by compacted chromatin. Chromatin is a macromolecular complex formed by DNA and histones. Several chemical modifications [in the schematics, only methylation (Me), acetylation (Ac), and ubiquitination (Ub) are shown] affecting histone tails and DNA cytosines modulate the level of chromatin compactness. (B) A simplified version of the DH induction. Firstly, haploid individuals are obtained either through crossing with haploid inducer mutants or through *in vitro* regeneration from haploid tissues. Finally, the whole genome is duplicated. This process can occur spontaneously or can be induced with chemicals such as colchicine. Horizontal red bars represent regions with methylated DNA, which change throughout the DH creation. For simplicity, only one chromosome pair is shown. 2n, H, and DH stand for a diploid with *n* chromosomes, haploid, and doubled haploid, respectively. Figure designed using Microsoft PowerPoint for Microsoft 365.

Developed during the second half of the 20th century, the induction of DHs was a breakthrough in crop breeding, allowing breeders to significantly speed up the process of obtaining pure homozygous lines, in great contrast to the classic backcrossing and selection process. DHs can be obtained in one generation, while conventional breeding requires 5–8 generations (Hooghvorst and Nogues, 2021). The first step to obtain DHs is to generate a haploid individual either by *in vitro* culture of gametophytic tissue or by crossing with mutant lines that induce the formation of haploid embryos (Gilles *et al.*, 2017). After the generation of haploids, chemical treatments with colchicine or oryzalin induce genome duplication to recover the normal ploidy levels (Fig. 1B).

Even though plants are known to be much more plastic than mammals in response to ploidy modifications (Scholes and Paige, 2014), it was largely presumed that DH technology may affect the chromatin environment and thus gene expression as a by-product of two sequential changes in ploidy levels. Using a haploid inducer line to remove the variability coming from the *in vitro* haploid induction, Piskorz *et al.* (2023) have carefully studied changes in DNA methylation during the DH generation process. Firstly, they generated three independent DH populations and compared the DNA methylation with the initial wild type. Not surprisingly, the authors found several thousands of differentially methylated regions (DMRs) between the different DH populations and the starting diploid Col-0. Strikingly though, most of those DMRs were found to be different among DHs. These changes in the chromatin environment appeared randomly but were clearly produced by DH induction. Aiming to dissect what is the origin of these DMRs, they compared side-by-side the DMRs at the haploid stage so they could assign the relative number of changes to each step. Interestingly, they found out that the induction of haploids mainly affected non-CG methylation, with substantial loss of CHH methylation in euchromatic regions.

In contrast, the chemical induction of whole-genome duplication (WGD) accounted for most of the CG and ~80% of the CHG DMRs, thus highlighting the impact of anti-mitotic treatments on symmetric DNA methylation. After comparison not only with the initial diploid but also with a spontaneous diploidization event, the authors could ensure a good correlation between symmetric DMRs and colchicine treatment.

One unexpected lesson from this work was that transposable elements (TEs), normally highly covered by DNA methylation, were barely changed at transcriptional levels after DH induction. As the authors discussed, albeit it can be phenotypically irrelevant in Arabidopsis, TE reactivation could have a harmful impact on crop performance. While this is a good sign that has to be confirmed in crops, it means that, indeed, a substantial proportion of the DMRs found directly affect gene body methylation and therefore their expression. The great variability among DH populations, ranging from 70 to 800 differentially expressed genes (DEGs), pointed once more to a fairly stochastic effect depending on the population analysed. DH technology is of course not the exclusive source of DMRs, which can emerge over time through mistakes during replication. However, according to the comparison made between DH creation and the random accumulation of DMRs over 30 generations (Becker *et al.*, 2011), it became clear that DH technology indeed enhances the number of DMRs and thus the downstream gene expression effects.

It is clear then that this approach randomly affects the chromatin environment. As claimed by the authors, the apparently stochastic effect can be used as an advantage to generate otherwise difficult to induce differentially methylated plants (epialleles), which is a nice molecular tool to understand epigenetic processes. However, we must not forget that the mechanisms explaining these undesired effects on chromatin are still unknown. This work is an important reminder of the relevance of a deeper understanding of the complex molecular mechanisms. These results based on whole-genome transcriptomics and DNA methylation analyses should prompt us to further dig into the effects of DH induction on chromatin rearrangement, whose effects very probably go beyond DNA methylation. Full profiles of histone modifications such as those related to DNA methylation (H3K9me2) or stable gene silencing (H3K27me3) should be combined with cell biology and the emerging single-cell sequencing technologies to address the implications of these effects in both model and crop systems. If these chromatin rearrangements are found to also occur in genetically complex crops, only a profound mechanistic understanding will help to tune down the potentially undesired side effects or redesign some of the steps.

Finally, but not less important, this piece of work pinpoints again the remarkable legal paradox between classic and modern breeding technologies. While random (i.e. uncontrolled) mutagenesis or classical interspecific breeding is allowed and considered safe worldwide, knowledge-driven technology such as GMOs or genome editing is considered potentially harmful and forbidden in a considerable part of the world including the European Union (Graham *et al.*, 2020). Established decades ago, DH technology is proven to be safe and substantially speeds up some breeding programmes, while it has long been suspected that the ploidy modifications may affect the chromatin status. Piskorz *et al.* (2023) have actually shown that DHs come with a certain amount of unpredictability, which to my knowledge has not represented any ethical or health issue. At least an equivalent amount of legal tolerance should be applied to newly developed approaches which by definition are considerably more targeted and whose side effects/off-targets have been thoroughly evaluated.
